# Two Small Molecule Drugs with Topical Applications, Diflunisal and Naphazoline, and Their Potentially Toxic Photodegradants: Analysis by Chemical and Biological Methods

**DOI:** 10.3390/molecules29174122

**Published:** 2024-08-30

**Authors:** Karolina Lejwoda, Anna Gumieniczek, Agata Filip, Beata Naumczuk

**Affiliations:** 1 Department of Medicinal Chemistry, Medical University of Lublin, Jaczewskiego 4, 20-090 Lublin, Poland; k.lejwoda94@gmail.com; 2 Cytogenetics Laboratory, Department of Cancer Genetics, Medical University of Lublin, Radziwiłłowska 11, 20-080 Lublin, Poland; agata.filip@umlub.pl; 3 Institute of Organic Chemistry, Polish Academy of Sciences, Kasprzaka 44, 01-224 Warsaw, Poland; beata.naumczuk@icho.edu.pl

**Keywords:** diflunisal and naphazoline, photodegradation, pH impact, phototoxicity, chromatography, in silico analysis, in vitro tests

## Abstract

Because of their topical application in patients and meaningful UV/VIS absorptive properties, the degradation and potential toxicity under irradiation of diflunisal (DIF) and naphazoline (NAF) were studied. In addition, the impact of pH on their photostability was examined, showing the highest degradation of acidic DIF at pH 1 and 13 and the highest degradation of basic NAF at pH below 7. An LC–UV analysis and chemical tests showed the first-order kinetics for their degradation and generation of reactive oxygen species (ROS). A UPLC-HRMS/MS analysis allowed us to identify four degradants of DIF (from DD-1 to DD-4) and six degradants of NAF (from ND-1 to ND-6). When Toxtree software was used, a high class III of toxicity was observed for DD-2, DD-3, and DD-4, and for all the NAF degradants. Furthermore, the ND-2 product, i.e., 2-[(1-methylnaphthalen-2-yl)methyl]-4,5-dihydro-1H-imidazole, was shown to present medium mutagenic and high tumorigenic effects according to OSIRIS Property Explorer. In addition, two in vitro tests on BALB/c 3T3 mouse fibroblasts showed a phototoxic effect of DIF and NAF at the lowest concentrations tested, i.e., 5 µg/mL. Thus, our present results could be useful to design further phototoxicity studies for DIF and NAF to minimize the risk of phototoxicity due to their photodegradation.

## 1. Introduction

**Figure 1:**
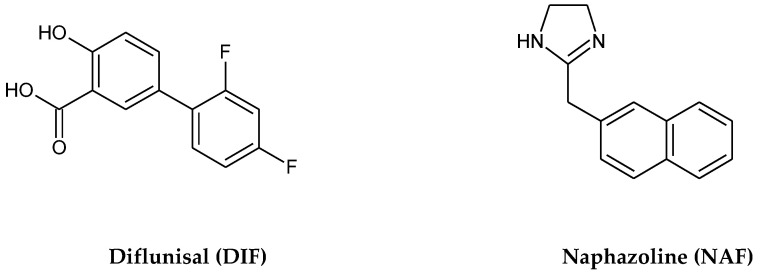
Chemical structures of diflunisal (DIF) and naphazoline (NAF).

Diflunisal (DIF), i.e., 2′,4′-difluoro-4-hydroxy-(1,1′-biphenyl)-3-carboxylic acid), is a salicylic acid derivative (Figure 1) used for the treatment of pain and inflammation, especially those associated with osteo- and rheumatoid arthritis. The drug can be given to patients orally, but it is most often applied topically on skin, which could lower the side effects related to oral administration [1,2]. In addition, new, important activities of DIF were proven recently, i.e., the treatment of transthyretin polyneuropathy and cardiac amyloidosis [3]. On the other hand, some limits, e.g., its poor water solubility and poor bioavailability, as well as its photosensitivity and potent phototoxicity, have been reported in the literature [4,5,6]. Thus, deeper knowledge of the photostability of DIF is necessary to minimize risk for patients due to the toxic properties of its photodegradation products.

Naphazoline (NAF), i.e., 2-(1-naphthylmethyl)-2-imidazoline, is a sympathomimetic agent which belongs to the imidazole derivative group (Figure 1). It is widely used in otolaryngology as a vasoconstrictor to treat rhinitis and in ophthalmology to relieve redness and itching of the eyes [7]. On the other hand, it was experimentally shown that NAF is photosensitive and its photolytic products could react with DNA [8,9,10].

The problems related to the photostability of pharmaceuticals are important in the context of their exposure to light after their topical application on the skin or to the eyes in patients. They are also important for the pharmaceutical industry, as far as the manufacturing and storage of such sensitive pharmaceuticals are concerned. Generally, knowledge of the photoreactivity of drugs can improve the design of more stable active pharmaceutical substances or final formulations with effective protection against light. It can also contribute by complementing official pharmacopeial monographs by introducing limits for risky photodegradation products present in pharmaceuticals [11]. Thus, experimental photodegradation is an important part of the drug development processes, including the identification of degradants and their potential toxicity. As a consequence, chemical tests, in silico as well as in vitro, are recommended to examine potential toxic effects due to the photodegradation of drugs [12]. 

Our present experiments were performed while bearing in mind the two perspectives mentioned above, i.e., the risk of the photodegradation of active substances in medicinal products and the risk of potential phototoxicity for patients due to toxic degradation products. The primary reasons for choosing DIF and NAF as the subjects for our experiments were their ability to absorb light in the UVA range and information from the literature on their potential phototoxic effects [4,8,9,13,14]. The lack of detailed studies, including forced-photodegradation studies and in vitro studies on their potential phototoxicity, was also taken into account.

Pharmaceutical technology often requires the use of different excipients that change the pH of the microenvironment of the final product, which can affect active substances, especially those with acid–base properties. DIF and NAF belong to different pharmacological classes, but both are characterized by at least one protonation site. DIF has an acidic pKa of 2.69, while NAF presents a basic pKa of 10.19 [15]. As a consequence, their different forms, ionized or non-ionized, can be present in solutions of different pH. Meanwhile different behaviors of drugs can be observed under UV/VIS irradiation, depending on their presence in the environment, as well as in the microenvironment [16,17]. The anti-inflammatory drug ketoprofen is one of the most interesting cases in this regard; the dominant role played by pH in its photochemistry has been proven [9]. 

Therefore, we decided to examine and compare the behavior of DIF and NAF, which may be sensitive to degradation due to their light-absorbing properties, which, in turn, may vary according to their acid–base properties. We believe that this comparison will broaden the scope of our research and could be interesting from both scientific and practical points of view.

The literature on the photodegradation of DIF in pure form or in formulations on the one hand, and its phototoxicity in patients on the other, is rather scarce. Only a few papers on the degradation of DIF, including photolytic conditions, were found [4,13,14]. However, in a previous study, the lysis of red blood cells was observed as a result of photosensitization by DIF. The authors proposed a mechanism involving reactive oxygen species (ROS) generation, i.e., superoxide anions (O_2_^−^) and singlet oxygen (^1^O_2_) [4]. As a consequence, solid dispersions of DIF with different polymer excipients were proposed for the manufacturing of topical pharmaceuticals in which the susceptibility of DIF to photodegradation could be lowered [2,5]. As far as NAF is concerned, two papers were found in the literature concerning its degradation, including the photolytic conditions [18,19]. In addition, its potential phototoxicity was investigated by studying its reactivity toward DNA. It was shown that the transient species produced under photolysis can react with DNA and promote its breakage under both anaerobic and aerobic conditions [8,9]. 

Thus, a detailed investigation of the photostability of these two small molecule drugs, i.e., acidic DIF and basic NAF, was performed in solutions at a wide pH range (1–13), using irradiation in the range 300–800 nm. Quantitative LC-UV and qualitative UPLC-HRMS/MS analyses to examine their degradation kinetics and to identify their photodegradation products were elaborated, together with chemical tests for the detection of ROS. Finally, in silico and in vitro methods were applied to estimate their potential phototoxicity.

## 2. Results and Discussion

### 2.1. Experimental Steps

First, a UV/VIS spectrophotometric analysis was performed to initially assess the risk of DIF and NAF photodegradation. Then, acidic DIF and basic NAF were irradiated with UVA/VIS light in solutions of pH 1–13 to compare their stability in terms of their ionization. The LC-UV experiments were carried out for a quantitative estimation of the degradation of DIF and NAF and to obtain kinetic measurements. The next steps of our study were focused on the identification of degradation products of DIF and NAF using UPLC-HRMS/MS analysis and on predictions of their toxicity using OSIRIS Property Explorer and Toxtree methods [20,21]. In addition, the possible generation of ^1^O_2_ and O_2_^−^ under the exposure of DIF and NAF to UVA/VIS irradiation was assessed using appropriate chemical tests with spectrophotometric measurements [22,23,24]. Finally, the phototoxic potential of the drugs was examined using in vitro methods (MTT, NRU and Live/Dead tests) on the cultured mouse BALB/c 3T3 fibroblasts.

### 2.2. UV/VIS Analysis

**Figure 2:**
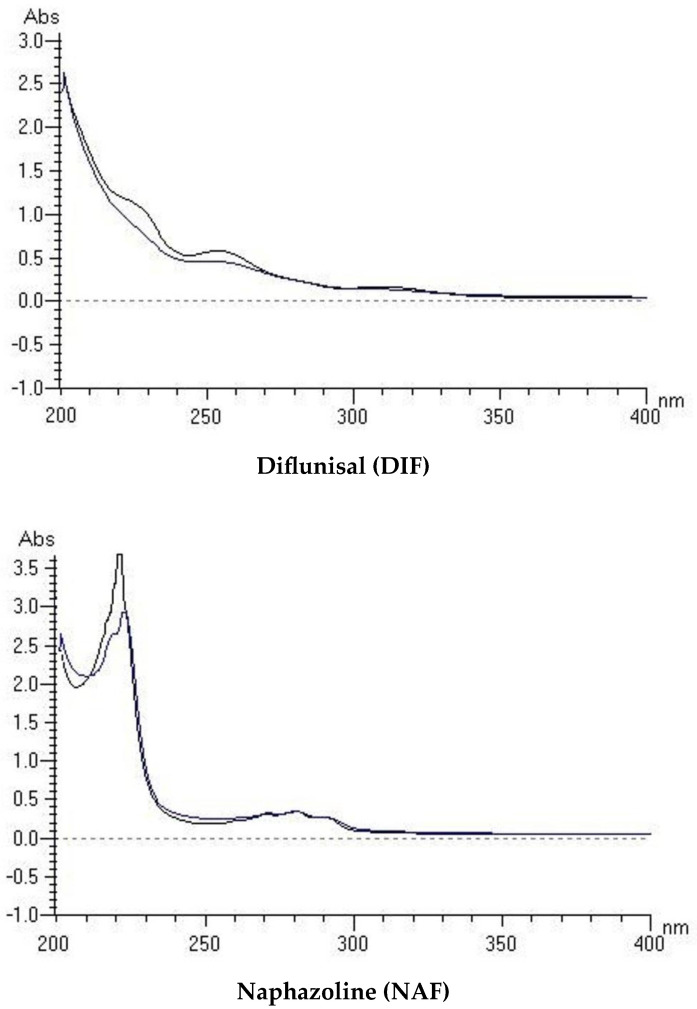
Spectra of diflunisal (DIF) and naphazoline (NAF) in a buffer of pH 7 after UVA/VIS irradiation with energy 2025 kJ/m^2^ (blue lines) in comparison with non-irradiated samples (black lines).

A UV/VIS analysis for DIF and NAF in solutions at a concentration 20 µM was carried out over the spectral range of 200–700 nm. When the spectra of methanolic solutions were analyzed, DIF showed two important peaks below 400 nm with a maximum at 254 nm, with the molar extinction coefficient (MEC) equal 29,200 M^−1^ cm^−1^, and at 309 nm, with MEC equal 8250 M^−1^ cm^−1^. On the other hand, NAF showed a meaning maxima at 223 nm with high absorbance >2 and at 284 nm with MEC equal 15,000 M^−1^ cm^−1^. Based on these results, i.e., MEC values higher than 1000 M^−1^ cm^−1^ near 300 nm, both of the drugs were considered to be photoreactive [22]. What is more, after irradiation, the spectra of DIF and NAF showed changes in the peak shapes compared to non-irradiated samples (Figure 2).

### 2.3. Parameters of LC-UV Method

The chromatographic conditions, as well as the linearity, limit of detection (LOD), limit of quantitation (LOQ), and the precision and accuracy of our LC-UV method, were evaluated and validated according to respective ICH guidelines [25]. The optimal conditions for chromatographic measurements of DIF and NAF, and the results obtained from validation steps, are shown in Table 1.

### 2.4. Impact of pH on Photodegradation of DIF and NAF

Two small molecule drugs, i.e., acidic DIF and basic NAF, were selected as the subjects of our present research in order to assess their photodegradation in environments with different pH. It needs to be highlighted that previous papers from the literature did not focus on the role of pH relative to their photochemical behavior, i.e., no respective experimental studies had previously been performed. DIF and NAF present different pharmacological and chemical properties, but both are characterized by at least one protonation site. On this basis, a different photobehavior can be expected depending on the neutral or ionized forms present in environments of varying pH. What is more, an interesting example of acidic ketoprofene from anti-inflammatory drugs was reported, where pH played the dominant role in its photochemistry, with the highest degradation observed for its acidic (non-ionized) form [9].

In the present study, the photodegradation of both DIF and NAF was estimated after exposure to UVA/VIS light (300–800 nm) for 7–35 h with energies of 18,902, 37,804, 56,706, 75,608 and 94,510 kJ/m^2^, using a solar simulator with a built-in meter. As a result, a degradation of 52.45–99.05% was observed for DIF after irradiation with a maximal dose of energy, depending on the pH value. As far as NAF is concerned, a percentage degradation ranging from 14.74% to 96.47% was noted. The kinetic calculations were based on the linear relationships between the concentrations of non-degraded DIF and NAF remaining after each irradiation, or the logarithms of these concentrations and the corresponding time of irradiation [26]. Using our experimental data, respective equations y = ax + b and the determination coefficients R^2^ were obtained for all the pH values. As a result, the plots based on the logarithms of concentrations showed stronger correlations, i.e., higher R^2^ values than the plots based on the concentrations, confirming the first-order kinetics for these degradation processes. Thus, further kinetic parameters, i.e., the degradation rate constant (k), the degradation time of 10% substance (t_0.1_), and the degradation time of 50% substance (t_0.5_), were calculated using the formula for this order kinetics.

**Figure 3:**
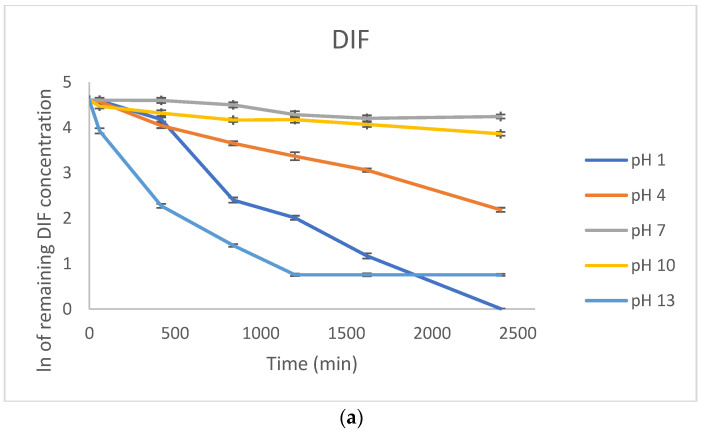

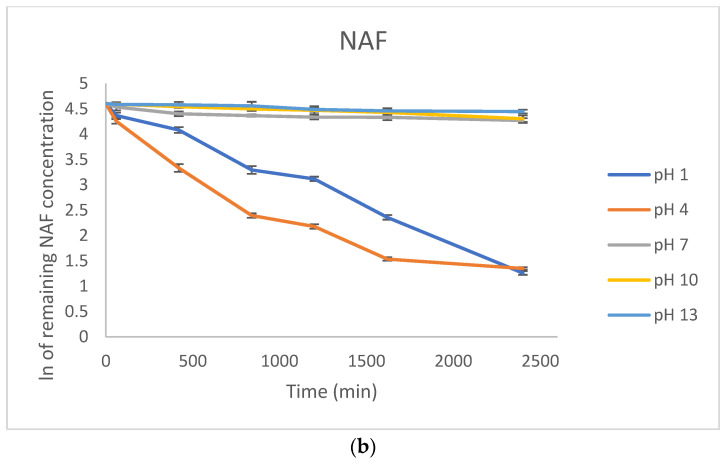
First-order plots of photodegradation of (**a**) diflunisal (DIF) and (**b**) naphazoline (NAF) in solutions of pH 1–13; the results are means for three measurements ± SD.

The impact of pH on percentage photodegradation of DIF and NAF, and kinetic parameters obtained for the first order reactions are shown in Figure 3 and Table 2.

In the literature, DIF was shown to degrade by 10% in an acidic medium (1 M HCl) and remain stable in alkaline conditions (1 M NaOH). However, the photodegradation of DIF was examined to a limited extent, i.e., in a solid state solely, where a degradation of 9% was observed [13]. The stability of DIF was also studied by exposing a methanolic solution to direct sunlight or UV irradiation at 254 and 366 nm. Then, DIF was observed to remain stable under direct sunlight and degraded by 58.72% under UV light [14]. The present study, however, was focused on degradation in solutions of different pH. The sensitivity of DIF to light was confirmed over the entire pH range with extrema at pH 1 and 13 and rate constants at levels of 10^−3^–10^−4^ s^−1^ (Table 2).

In a study from the literature, NAF was shown to be degraded up to 78.5% when 5 M HCl was used, up to 44.9% when 5 M NaOH was used, and up to 76.0% under oxidative conditions. The drug was also exposed to photolytic conditions; however, only basal energies recommended for official confirmatory ICH tests were applied, i.e., 200 W/m^2^ and 1,200,000 luxh [27], in which NAF did not show visible degradation [16]. In another study from the literature, NAF was shown to be degraded above 50% in 1 M NaOH but rather stable as a solid substance exposed to UV light at 254 nm [18]. In the present study, sensitivity of NAF to light was studied in forced conditions and lower or higher degradation of NAF was shown over the entire pH range with extrema at pH 1–4 and degradation above 90% and rate constants of 10^−3^ s^−1^. However, photodegradation at pH ≥7 showed k values at a level of 10^−5^ s^−1^, and, as a consequence, the t_0.1_ and t_0.5_ values were 50–250 times longer than at pH 1–4. Finally, NAF showed the lowest photodegradation (14.74%) in its non-ionized form in alkaline conditions at pH 13 (Table 2). In the literature, the photochemical behavior of NAF in its protonated and unprotonated (basic) forms was explored theoretically. The authors proposed that the molecular orbital distributions in NAF may quench excitation in its basic structure. On the other hand, the naphthalene to imidazoline charge transfer upon HOMO−LUMO excitation in the protonated form may occur, resulting in greater singlet oxygen generation [9,10].

**Figure 4:**
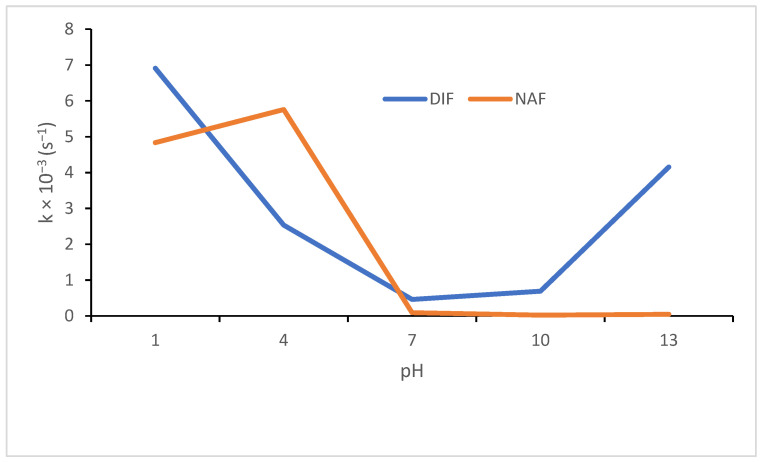
The degradation of diflunisal (DIF) and naphazoline (NAF) in solutions of different pH, under UV/VIS irradiation with energy 94,510 kJ/m^2^; k = the rate constant of the first order reactions.

Thus, our study clearly showed the impact of pH on the photodegradation of drugs, with the highest degradation of acidic DIF at pH 1 and 13 and the highest degradation of basic NAF at pH below 7 (Figure 4). As a result, the need to select an optimal microenvironment for the manufactured formulations of DIF and NAF could be postulated, taking into account their chemical properties and susceptibility to degradation, depending on their degree of ionization.

The pH range used in the present experiments was wider than that typically used in the pharmaceutical industry for the confirmation of drug stability or after the administration of drugs in patients. Nevertheless, it is often necessary to use extreme pH to achieve a sufficient solubility of active substances; then, the resultant pH can lead to drug instability [28]. What is more, the ICH Q1A(R2) guidelines recommend evaluations of the photolysis and hydrolysis of drugs over a wide range of pH [29]. Therefore, we decided to perform our experiments under forced degradation conditions, also using extreme pH, in order to obtain as many photodegradation products as possible. In addition, we wanted to compare the photostability of two drugs with different acid–base properties, i.e., acidic DIF and basic NAF, providing their k versus pH profiles over a wide pH range.

### 2.5. Identification of Degradation Products by LC-HRMS Method and In Silico Analysis

After UVA/VIS irradiation of DIF and NAF in respective solutions, the identification of their degradation products was performed using a basis of UPLC-MS analysis. The proposed structures were elucidated by fragmentation path analysis based on the HR fragmentation mass spectra. Finally, OSIRIS Property Explorer (https://www.organic-chemistry.org/prog; accessed on 20 April 2024) and Toxtree v3.1.0. software [20,21] were used to predict the class of toxicity or the mutagenic, tumorigenic, irritant, and reproductive effects for the identified degradation products. It should be emphasized that this part of our paper brings completely new information to the literature concerning DIF and NAF, because, as was stated above, such results have not been reported previously.

#### 2.5.1. Identification of Degradation Products of DIF

**Figure 5:**
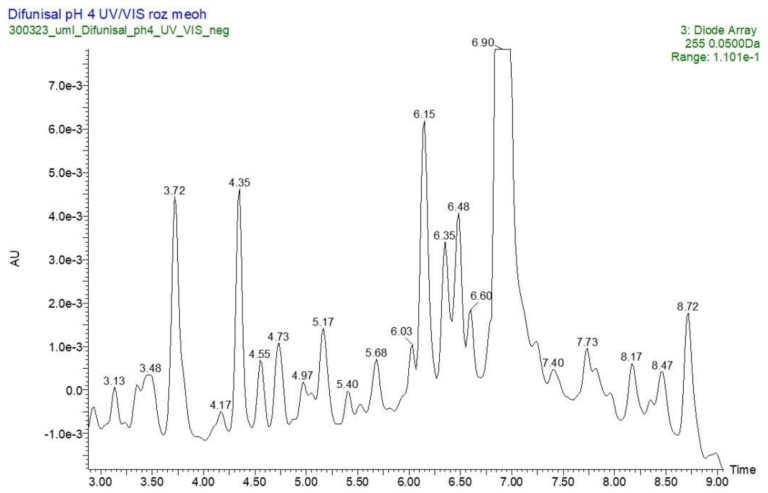
A UPLC-UV chromatogram at 255 nm of diflunisal (DIF) with t_R_ = 6.90 min and its degradation products (t_R_ = 3.72, 5.68, 6.15 and 6.39 min) after UVA/VIS irradiation at pH 4.

As far as the DIF analysis is concerned, a representative UPLC-UV chromatogram for the sample of DIF irradiated in solution at pH 4 is presented in Figure 5, where t_R_ = retention time.

**Figure 6:**
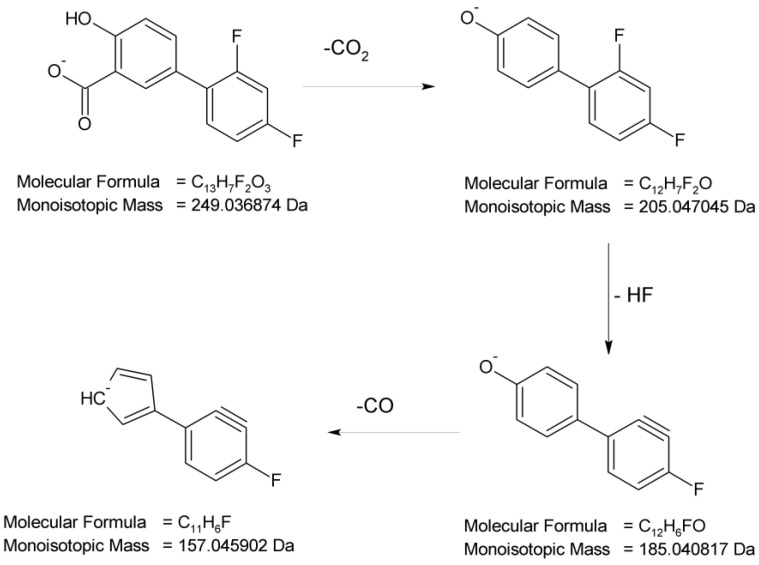
Proposed fragmentation pattern of diflunisal (DIF).

The full scan mass spectra in a negative ion mode were obtained for DIF, in which the deprotonated molecule at *m*/*z* 249 was detected. Then, the MS/MS spectrum showed the product ion at *m*/*z* 205 due to the elimination of CO_2_ from the parent molecule that formed subsequent ions at *m*/*z* 185 (the loss of HF molecule) and 157 (subsequent elimination of CO). The proposed fragmentation pattern for DIF is presented in Figure 6.

**Figure 7:**
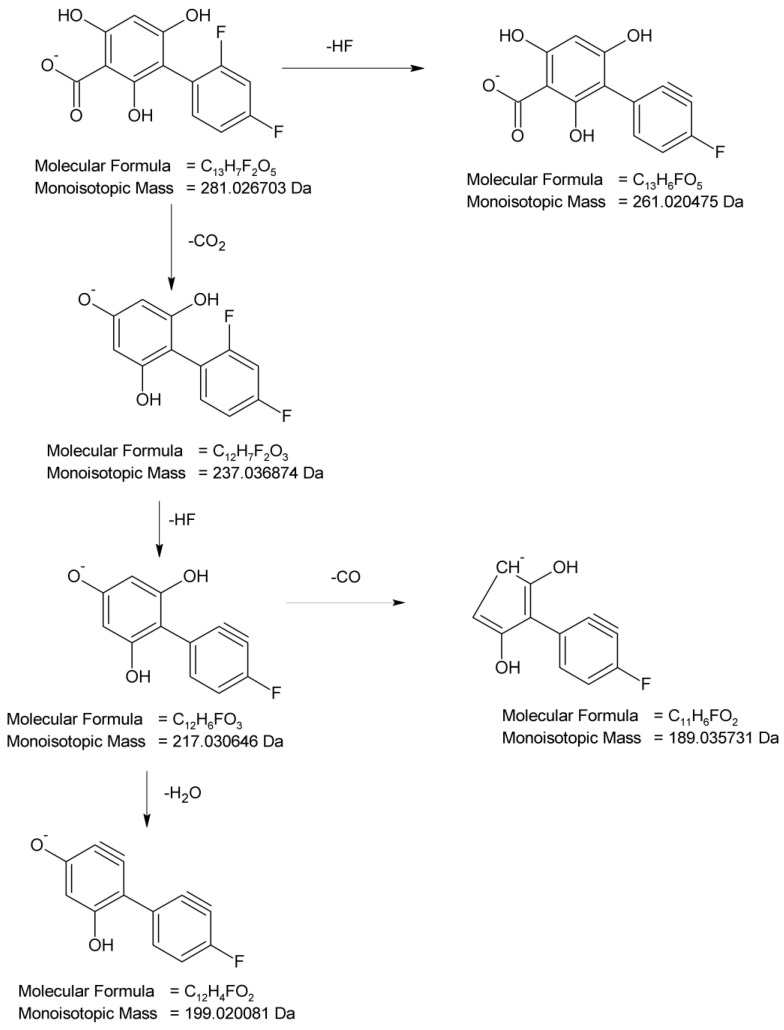
Proposed fragmentation pattern of DIF degradation product 1 (DD-1).

In the present study, four degradation products of DIF from DD-1 to DD-4 were identified by analyzing the samples irradiated at pH 4. The full scan mass spectra of the peak with t_R_ = 3.72 min were obtained, in which the deprotonated molecule at *m*/*z* 281 was detected. This degradation product (DD-1) was formed by a double hydroxylation of the salicylic acid structure in the parent DIF molecule. Its MS/MS spectrum showed the product ion at *m*/*z* 237 formed after the elimination of CO_2_ and at *m*/*z* 261 after the elimination of HF. Next, the product at *m*/*z* 237 fragmented to a subsequent ion at *m*/*z* 217 through the elimination of HF that further formed the ion at *m*/*z* 199 due to dehydroxylation, or the ions at *m*/*z* 189 due to the loss of CO and further at *m*/*z* 173 due to subsequent dehydroxylation. As a consequence, the analysis of this fragmentation pattern, accurate mass measurements, and the elemental composition indicate the structure of DD-1 as 2′,4′-difluoro-2,4,6-trihydroxy [1,1′-biphenyl]-3-carboxylic acid (Figure 7). 

**Figure 8:**
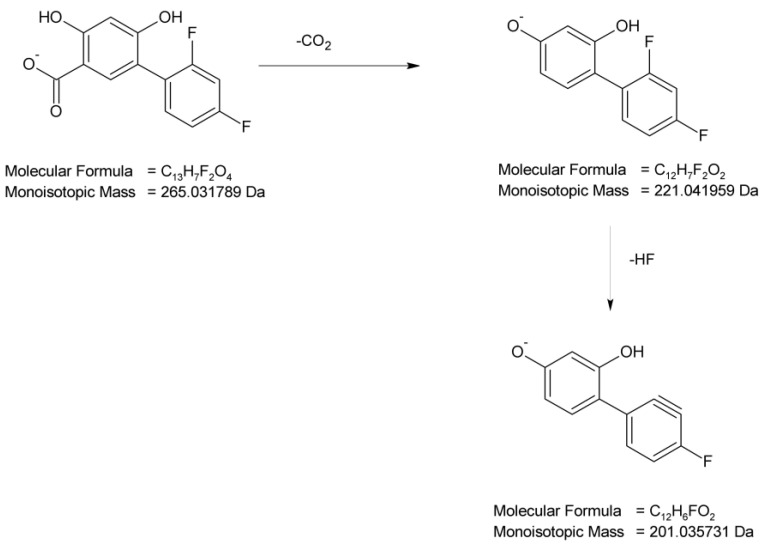
Proposed fragmentation pattern of DIF degradation product 2 (DD-2).

The next degradant generated via the irradiation of samples of DIF at pH 4, i.e., DD-2 with *m*/*z* 265, was formed by a single hydroxylation of the salicylic acid structure in the parent molecule. The subsequent fragmentation step was similar to DD-1, i.e., the elimination of CO_2_ (*m*/*z* 221) and the elimination of HF (*m*/*z* 201), which led to the proposed structure of DD-2 as 2′,4′-difluoro-4,6-dihydroxy[1,1′-biphenyl]-3-carboxylic acid (Figure 8).

**Figure 9:**
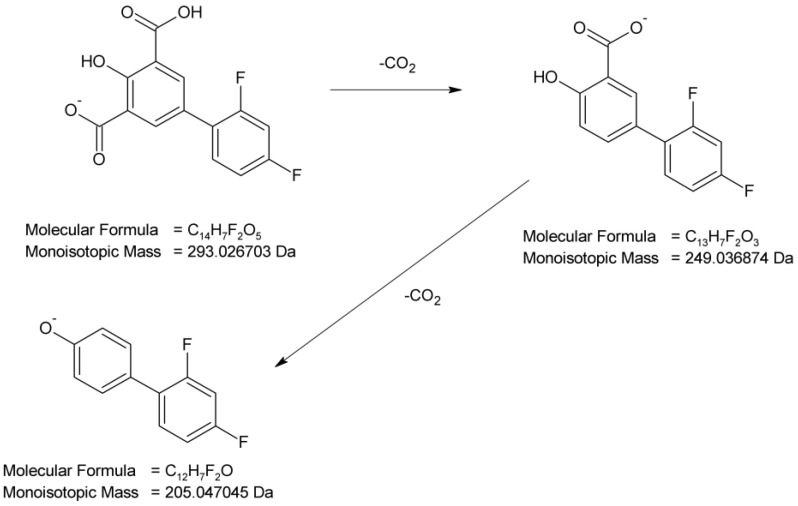
Proposed fragmentation pattern of DIF degradation product 3 (DD-3).

As far as DD-3 (*m*/*z* 293) is concerned, the carboxylation of the salicylic acid structure in the parent drug molecule is proposed. The MS/MS spectrum of its molecular ion showed two product ions which were similar to the fragmentation patterns described above, i.e., the elimination of CO_2_ (*m*/*z* 249) leading to the parent drug molecule and further decarboxylation (*m*/*z* 205). The analysis of this fragmentation pattern from the MS/MS spectrum (Figure 9), accurate mass measurements, and the elemental composition indicate the proposed structure of DD-3 as 2′,4′-difluoro-4-hydroxy[1,1′-biphenyl]-3,5-dicarboxylic acid.

**Figure 10:**
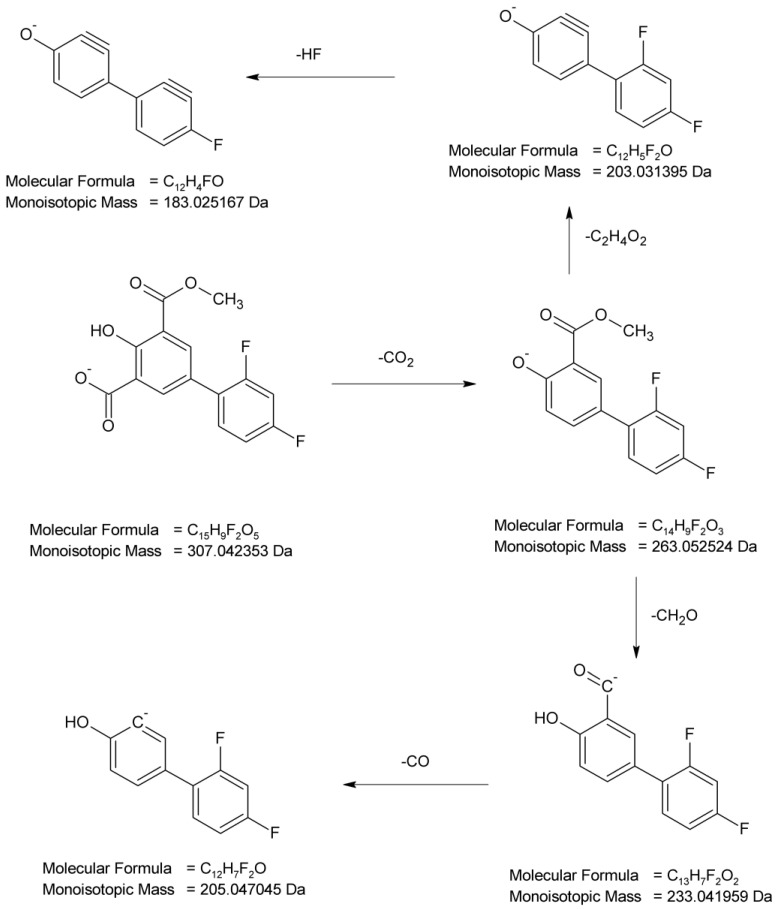
Proposed fragmentation pattern of DIF degradation product 4 (DD-4).

As far as DD-4 (*m*/*z* 307) is concerned, the carboxylation of the salicylic acid structure in the parent drug molecule and the subsequent esterification in the presence of methanol is proposed. The MS/MS spectrum of its molecular ion showed the product ions, which were similar to the fragmentation patterns described above, i.e., the elimination of CO_2_ leading to ions at *m*/*z* 263 and 205. The ion at *m*/*z* 263 could be degraded by the elimination of the ester group (*m*/*z* 203) and the further elimination of HF (*m*/*z* 183). The ion at *m*/*z* 263 could also degrade by the elimination of the ester group (*m*/*z* 233) and the further elimination of CO (*m*/*z* 205). The analysis of this fragmentation pattern from the MS/MS spectrum (Figure 10), accurate mass measurements, and the elemental composition indicate the proposed structure of DD-4 as 2′,4′-difluoro-4-hydroxy-5-(methoxycarbonyl)[1,1′-biphenyl]-3-carboxylic acid.

**Figure 11:**
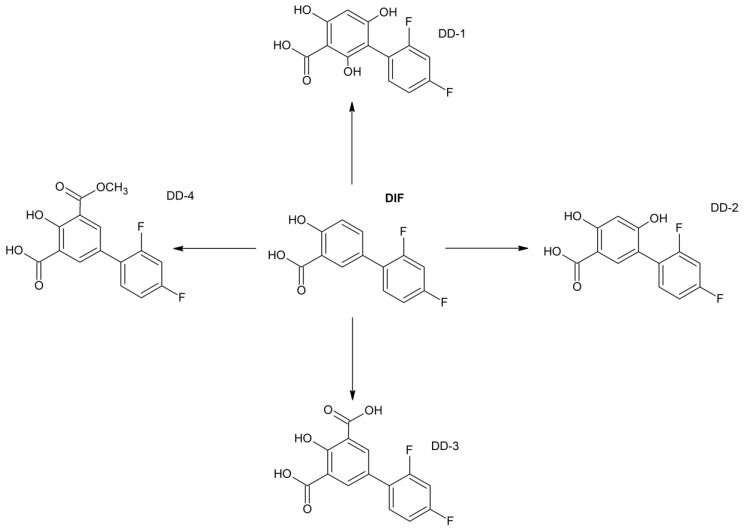
Proposed photodegradation products of diflunisal (DIF) at pH 4.

All things considered, the photodegradation of DIF generally occurred by the hydroxylation of the salicylic acid moiety in the parent drug molecule and carboxylation or carboxylation, with the subsequent esterification in the presence of methanol (Figure 11). At the same time, the observed processes were proved to be the light-induced, because none of these degradants was detected in the non-irradiated solutions at an identical pH. Previous studies from the literature demonstrated that DIF undergoes photodefluorination, leading to the formation of the main degradation product described as 2′-(2‴,4‴-difluoro-3″-carboxy-[1″,1‴-biphenyl]-4″-oxy)-4′-fluoro-4-hydroxy-[1,1′-biphenyl]-3-carboxylic acid. As was also mentioned, this compound was found to be responsible for the photohemolysis of red blood cells in subsequent biological tests [4,6]. In our study, the elimination of orto-fluoride atoms was shown in the fragmentation patterns of the parent DIF, DD-1 and DD-4 compounds (Figure 6, Figure 7 and Figure 10).

When in silico methods for toxicity assessment were used, i.e., OSIRIS Property Explorer (https://www.organic-chemistry.org/prog; accessed on 20 April 2024) and Toxtree v3.1.0 [20,21], some interesting results were obtained regarding the DIF degradation products. The OSIRIS software predicts the toxicity risk connected with the specific fragments in a molecule. These potentially toxic fragments were chosen through an insightful study of compounds that are known to be toxic according to the Registry of Toxic Effects of Chemical Substances database (RTECS) [30]. Our in silico analysis of all DD structures did not show fragments with the risk of mutagenic, tumorigenic, irritant, or reproductive effects. On the other hand, the Toxtree software classified the compounds DD-2, DD-3 and DD-4 as belonging to class III, with high toxicity based on the Cramer rules, determined based on the structure-based Treshold of Toxicological Concern (TTC) and expected exposure [31].

#### 2.5.2. Identification of Degradation Products of NAF

**Figure 12:**
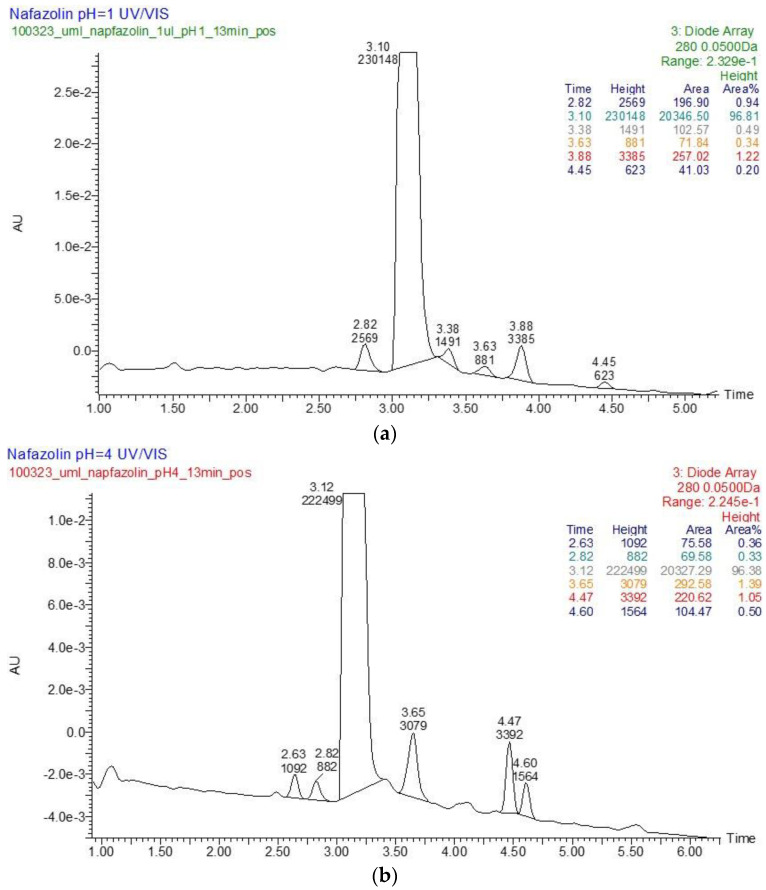
UPLC-UV chromatograms at 280 nm of naphazoline (NAF) with t_R_ = 3.10 min and its degradation products after UVA/VIS irradiation at pH 1 (**a**) and pH 4 (**b**).

In the literature, NAF was described as being liable to hydrolysis after refluxing with 1 M NaOH, giving 1-naphthalene acetic acid as a major degradation product [32]. However, there is not any information on its photodegradation products and their identification using the LC-MS/MS methods. In the present study, six degradation products of NAF (from ND-1 to ND-6) were identified by analyzing samples irradiated at pH 1–13. The representative UPLC-UV chromatograms of the samples irradiated in solutions of pH ≤ 4 are presented in Figure 12.

**Figure 13:**
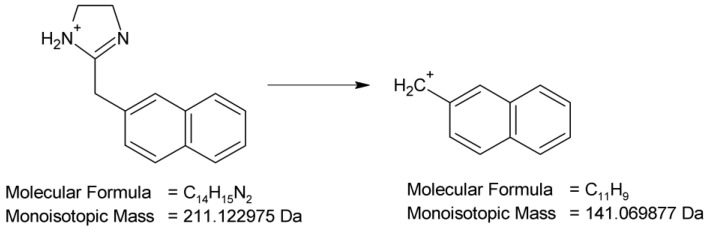
Proposed fragmentation pattern of naphazoline (NAF).

Full scan mass spectra in positive ion mode were obtained for NAF, in which the protonated molecule at *m*/*z* 211 was detected. Then, the MS/MS spectrum showed the product ion at *m*/*z* 141 due to the elimination of the imidazoline moiety from the parent NAF structure (Figure 13).

**Figure 14:**
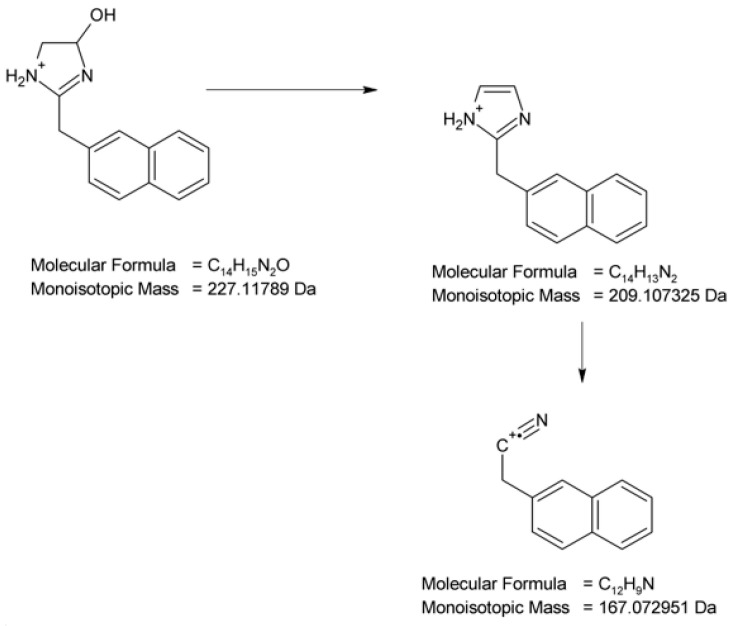
Proposed fragmentation pattern of NAF degradation product 1 (ND-1).

At pH 1–4, the photodegradation of NAF occurred by the hydroxylation of imidazoline ring and the methylation of naphthalene ring in the parent drug molecule. The mass spectra showed the protonated ions [M + H]+ with *m*/*z* 227 for ND-1 with t_R_ = 2.82 min and *m*/*z* 225 for ND-2 with t_R_ = 3.63 min (Figure 12). The product at *m*/*z* 227 produced a subsequent ion at *m*/*z* 209 due to oxidative dehydroxylation, and at *m*/*z* 167 due to the further opening of the imidazole ring. As a consequence, the analysis of this fragmentation pattern, accurate mass measurements, and the elemental composition indicate the structure of ND-1 as 2-[(naphthalen-2-yl)methyl]-4,5-dihydro-1H-imidazol-4-ol (Figure 14).

**Figure 15:**
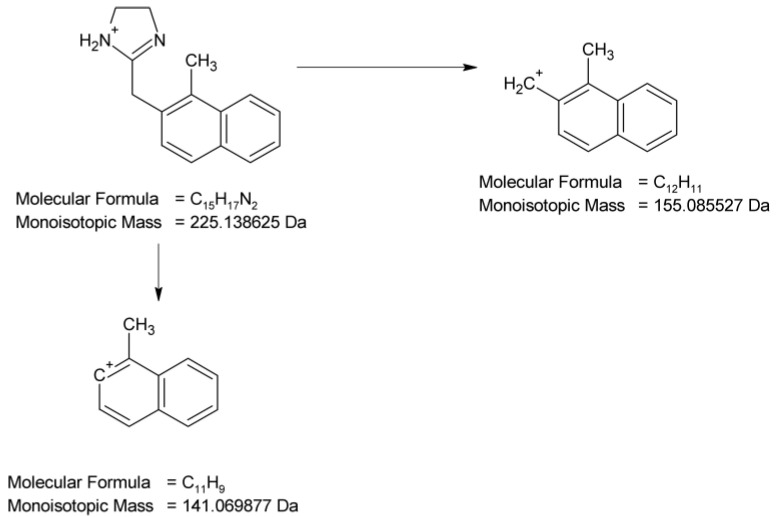
Proposed fragmentation pattern of NAF degradation product 2 (ND-2).

However, the product ion at *m*/*z* 225 was present due to the methylation of the naphthalene structure, while the subsequent elimination of imidazoline or the (naphthalen-2-yl)methyl group from the structure produced ions at *m*/*z* 155 and 141, respectively. It should be noted that under the described degradation conditions, the methylation of naphthalene rings can occur in different positions. However, we were not able to establish this methylation position based solely on this fragmentation pathway. One of the proposed structures of ND-2 is presented in Figure 15.

**Figure 16:**
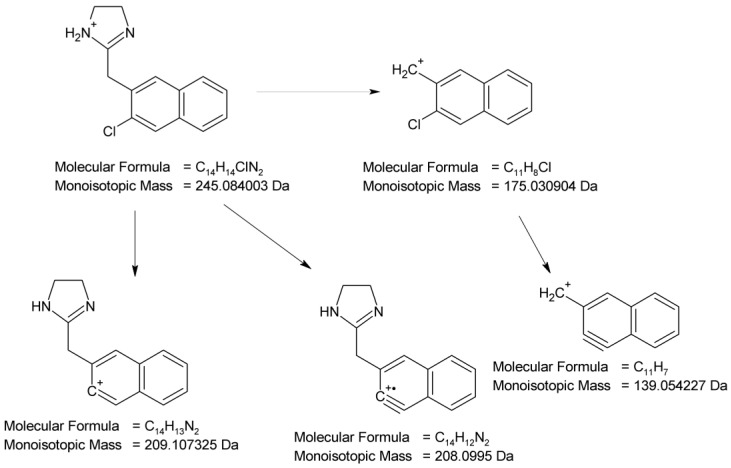
Proposed fragmentation pattern of NAF degradation product 3 (ND-3).

In the UPLC-UV chromatogram of the NAF sample irradiated at pH 1, we observed two peaks at t_R_ = 3.38 and 3.89 min (two isomers), whose MS spectra showed a molecular ion *m*/*z* 245 with an isotopic profile, suggesting the presence of a chlorine atom. This may imply that under these conditions, NAF, as a hydrochloride, undergoes a reaction resulting in two isomers of NAF substituted with a chlorine atom. An analysis of the respective fragmentation path allowed us to propose one of the possible structures of ND-3. As in the case of the previously mentioned methylation, we were unable to determine the exact location of the chlorine. The proposed structure of one of the isomers is shown in Figure 16.

**Figure 17:**
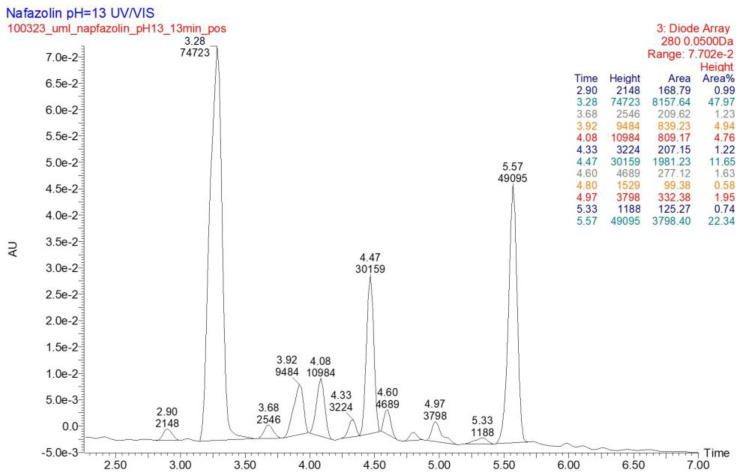
A UPLC-UV chromatogram at 280 nm of naphazoline (NAF) with t_R_ = 3.28 min and its degradation products after UVA/VIS irradiation at pH 13.

A representative UPLC-UV chromatogram of the samples of NAF irradiated in solutions of pH > 4 is presented in Figure 17.

**Figure 18:**
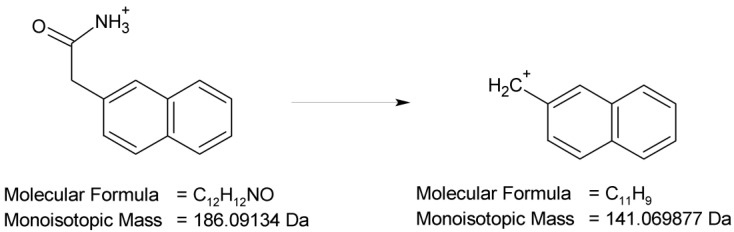
Proposed fragmentation pattern of NAF degradation product 4 (ND-4).

At pH 4–13, the photodegradation of NAF occurred by the opening of an imidazoline ring, producing an ion at *m*/*z* 186 with t_R_ = 4.47 min (Figure 16), and a subsequent ion at *m*/*z* 141. Thus, the structure of ND-4 is proposed as 2-(naphthalen-2-yl)acetamide (Figure 18).

**Figure 19:**
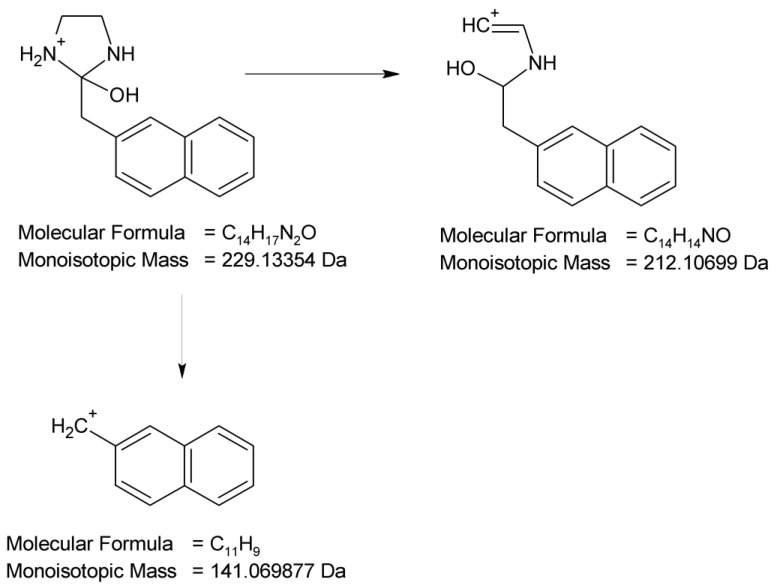
Proposed fragmentation pattern of NAF degradation product 5 (ND-5).

By analyzing the LC-UV-HRMS results of NAF after UVA/VIS irradiation at pH 13, it was possible to identify two further degradation products, i.e., ND-5, with t_R_ = 3.28 min, and ND-6, with t_R_ = 4.08 min (Figure 17). It was confirmed that the photodegradation of NAF can also occur by the hydroxylation or the hydroxylation and oxidation of imidazoline rings, producing ions at *m*/*z* 229 and 257. As far as ND-5 (*m*/*z* 229) is concerned, its MS/MS spectrum showed fragmentation patterns via the elimination of (naphthalen-2-yl)methyl groups or the elimination of NH_3_, leading to ions at *m*/*z* 141 or 212, respectively. Our analysis of the fragmentation pattern from the MS/MS spectrum, accurate mass measurements, and the elemental composition indicate the structure of ND-5 as 2-[(naphthalen-2-yl)methyl]imidazolidin-2-ol (Figure 19).

**Figure 20:**
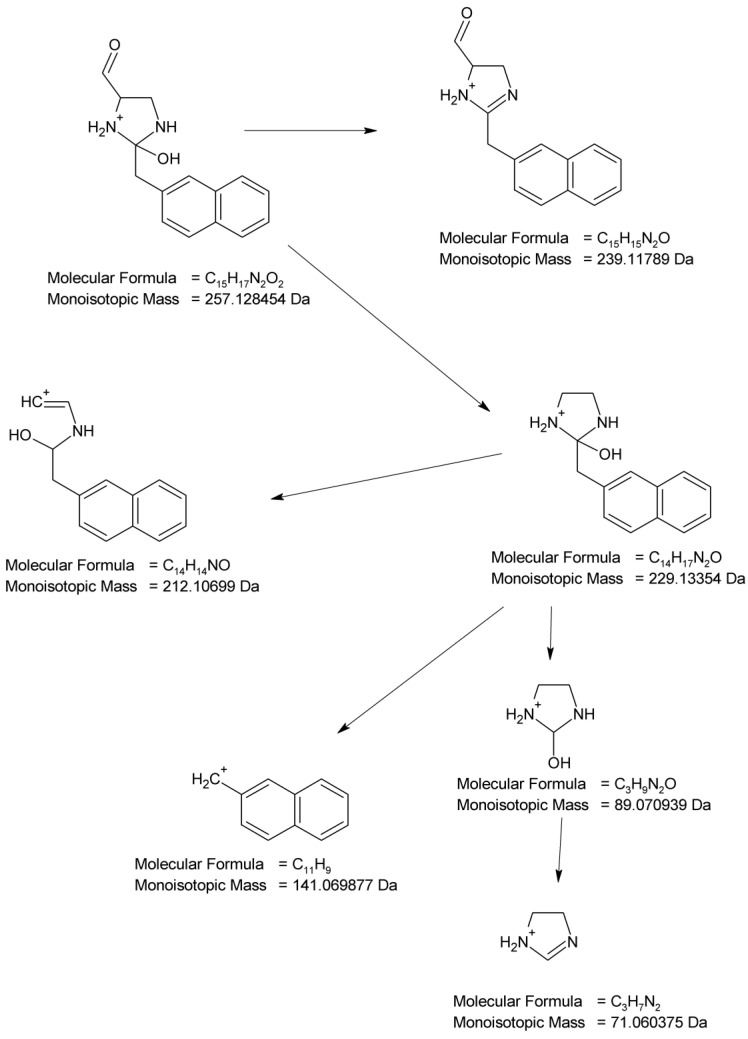
Proposed fragmentation pattern of NAF degradation product 6 (ND-6).

As far as ND-6 (*m*/*z* 257) is concerned, its MS/MS spectrum showed ions at *m*/*z* 239 and 229, i.e., ND-5, which implied the proposed structure of ND-6 as 2-hydroxy-2-[(naphthalen-2-yl)methyl]imidazolidine-4-carbaldehyde (Figure 20).

**Figure 21:**
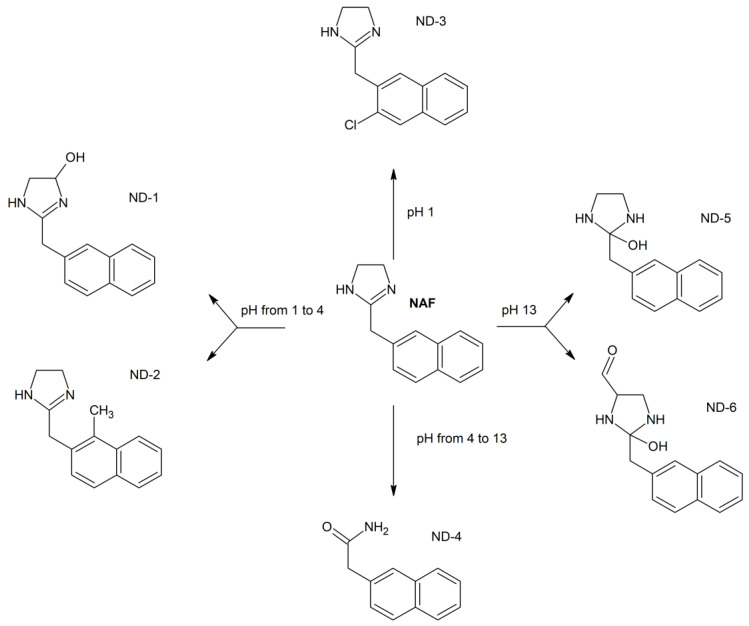
Proposed photodegradation products of naphazoline (NAF) under different pH conditions.

All in all, the photodegradation of NAF occurred mainly by the hydroxylation and oxidation of the imidazoline moiety and the methylation of naphthalene rings. Moreover, we could observe the product of the methylation and substitution of chlorine atoms in the naphthalene rings. Because these identified degradation products were not observed in the non-irradiated solutions of NAF at an identical pH, it could be proposed that the observed changes were due to the impact of light. All the identified degradation products from the samples of NAF irradiated at different pH are summarized in Figure 21.

Finally, in silico methods for toxicity assessments of all NAF degradants were used, which showed more variable results than those in the case of DIF. While OSIRIS Property Explorer did not show any toxicity risk for the ND-1, ND-3, ND-4, ND-5 and ND-6, it did show it for ND-2, i.e., 2-[(1-methylnaphthalen-2-yl)methyl]-4,5-dihydro-1H-imidazole, which was shown to present medium mutagenic and high tumorigenic effects. What is more, when the Toxtree software was used, a class III of toxicity was indicated for all degradation products, including ND-2.

Conducting phototoxicity studies in silico could serve as an additional source of information in relation to in vitro studies. In addition, these methods make it possible to select degradation products that present a higher risk of toxicity, e.g., photogenotoxicity. In the next step, we isolated these compounds from the degraded samples to confirm their structures using additive methods, like NMR and IR, and finally, we individually assessed their potential toxicity using in vitro methods [26].

Bearing in mind the two perspectives mentioned above, i.e., the risk of photodegradation of active substances in marketed formulations exposed to UV/VIS light and the risk of phototoxicity for patients due to toxic degradation products, at least some of our in silico results concern a pH range which can be found in the formulations or after their use by patients, e.g., pH 4–7. Using the Toxtree software, a high class of toxicity was obtained for DD-2, DD-3, and DD-4 from the samples of DIF irradiated at pH 4, as well as for all the NAF degradants, including ND-1, generated at pH 4, and ND-4, detected at pH from 4 to 7. What is more, ND-2 from the samples of NAF irradiated at pH 4 was shown to present medium mutagenic and high tumorigenic effects according to OSIRIS Property Explorer.

Nevertheless, our study has a few limitations. In silico analysis showed the need to use several screening programs to avoid missing any compound with an increased risk of toxicity. Since we utilized only two simple in silico methods for the prediction of toxicity, the present results may not be as reliable as those obtained with more comprehensive tools. However, our study showed that some degradation products present a higher risk than others, e.g., ND-2. Such compounds should be isolated from the degraded samples to individually assess their potential toxicity in vitro.

### 2.6. ROS Generation

The potential phototoxicity of drugs could be examined by monitoring singlet oxygen (^1^O_2_) and superoxide radical anion (O_2_^−^) generation under the light exposure using radiation doses based on official recommendations for phototoxicity testing, starting from 5–20 J/cm^2^ (50–200 kJ/m^2^). Generally, a substance is considered to be photoreactive when ^1^O_2_ values are ≥25 and/or O_2_^−^ values are ≥70 [22]. According to these criteria, both DIF and NAF could be assessed as being photoreactive with ^1^O_2_ values higher than 25 under irradiation with energy 234 kJ/m^2^ and less photoreactive with O_2_^−^ values higher than 70 under irradiation with energy 1350 kJ/m^2^ (Table 3).

What is more, the results obtained for DIF are in agreement with previous data from the literature, where the ROS generation under the exposure of DIF to light was confirmed [4,6]. As far as NAF is concerned, previous studies showed that ROS and nitrogen reactive species were produced upon laser excitation; these compounds were probably responsible for the observed DNA damage [8,9,10]. This prompted us to plan and conduct three in vitro tests with the cultured mouse BALB/c 3T3 fibroblasts as further experiments in order to better understand the potential toxic effects of DIF and NAF. Their topical application on skin or eyes in patients and their exposition to environmental light were also important reasons.

### 2.7. In Vitro Tests

For the MTT and NRU tests, the irradiation energy was set to 5 J/cm^2^ based on the official recommendations for in vitro phototoxicity testing [22]. The fibroblast viability was estimated in each group co-treated with DIF or NAF at concentrations increasing from 0 to 1000 µg/mL and UVA/VIS irradiation, in relation to non-irradiated cells incubated with similar concentrations of DIF or NAF. In the next steps, the viability of the fibroblasts was compared between the cells irradiated in the presence of DIF or NAF at concentrations of 5–1000 µg/mL and the respective control groups exposed to irradiation without drugs (0 µg/mL). Finally, a simultaneous determination of live and dead cells was performed using a Live/Dead test with two reagents, i.e., acetoxymethyl ester of calcein (CA-AM) and ethidium homodimer-1 (EthD-1), which reflect the intracellular esterase activity and membrane integrity, respectively. In live cells, calcein (CA) is produced from CA-AM by active esterases and then accumulates in the cytoplasm. On the other hand, EthD-1 penetrates damaged cell membranes and binds to nucleic acids in dead cells [33]. During this test, the fibroblasts were irradiated with an energy of 5 J/cm^2^ in the presence of DIF and NAF at a concentration 1000 µg/mL or without drugs (0 µg/mL).

**Figure 22:**
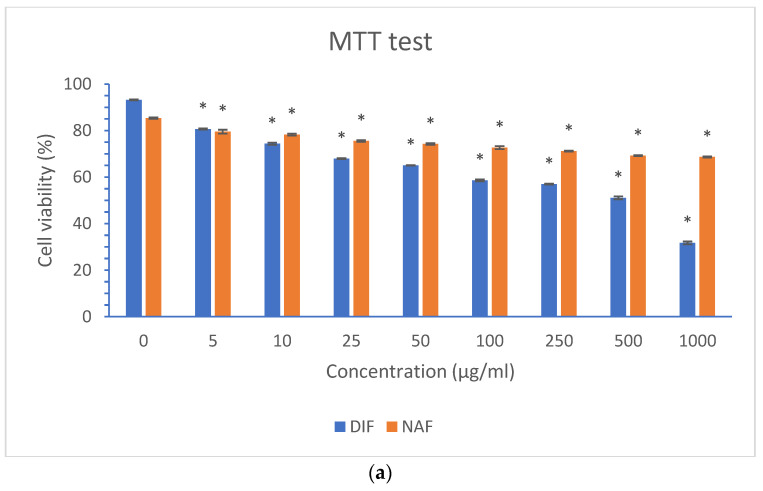

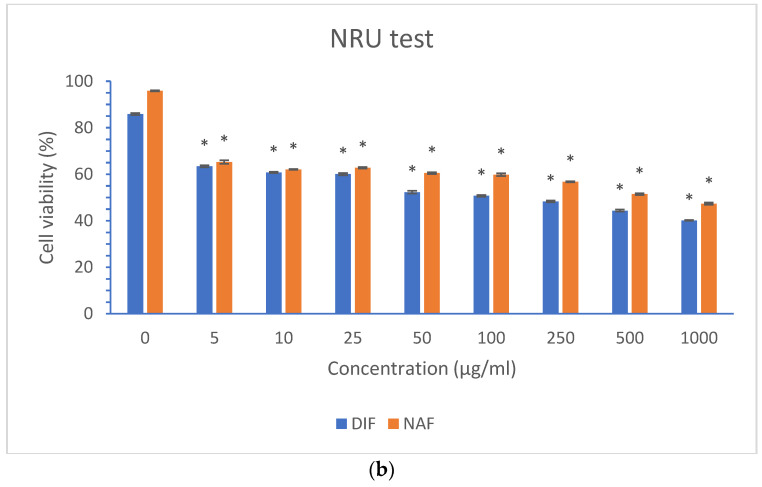
Fibroblast viability after irradiation with an energy of 5 J/cm^2^ and 0–1000 µg/mL of diflunisal (DIF) and naphazoline (NAF) in MTT test (**a**) and NRU test (**b**). The results are presented as mean ± SEM (standard error of the mean, n = 3). The DIF and NAF affect fibroblast viability, starting from a concentration of 5 µg/mL (* significant difference versus 0 µg/mL at *p* < 0.005).

As far as the DIF phototoxicity is concerned, the results of the MTT test showed that the viability of fibroblasts irradiated in the presence of DIF was in the range of 93.65–30.77%, with DIF concentrations increasing from 0 to 1000 µg/mL. A statistically significant (at *p* < 0.005) decrease in the cell viability was observed at 5 µg/mL, while the concentration at which the cell viability was reduced by approximately 50% was 500 µg/mL (Figure 22a). A similar drop in cell viability from 86.71 to 40.01% was observed with increasing DIF concentrations in our NRU test. A statistically significant impact of DIF was also observed starting from the concentration of 5 µg/mL. At the same time, fibroblasts irradiated with DIF at a concentration ≥ 100 µg/mL showed reduced cell viability by approximately 50% (Figure 22b).

**Figure 23:**
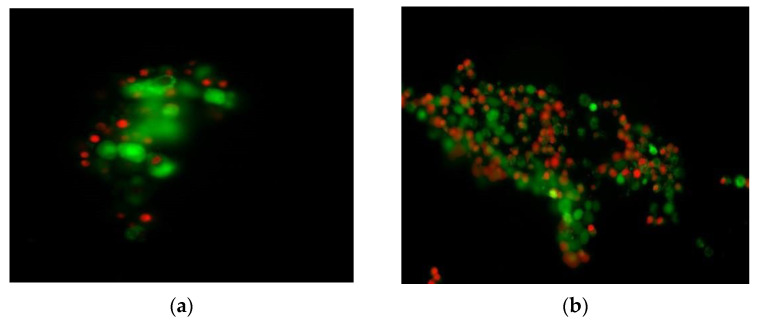
Representative photos obtained in Live/DEAD test with BALB/c 3T3 fibroblasts irradiated with UVA/VIS light in the presence of diflunisal (DIF) at concentration 1000 µg/mL (**b**) or without DIF (**a**), where the live cells are green and the dead cells are red.

It was also showed (Live/Dead test) that DIF at a concentration of 1000 µg/mL reduced the live cell number by about 10%, in comparison with the respective non-irradiated samples. The representative photos clearly showed a larger number of dead cells in the sample irradiated in the presence of DIF in comparison with the sample irradiated without DIF (Figure 23). As noted in the literature, one degradation product of DIF showed strong hemolytic activity. The authors of that study concluded that the generation of ROS, including ^1^O_2_ and O_2_^−^, could be involved in this photohemolytic proces [4]. Taking the above into account, i.e., the data from the literature and our present results from MTT, NRU, Live/Dead and ROS tests, we suggest the potential phototoxicity of DIF and the involvement of free radical generation in the observed effects.

Regarding the impact of irradiated NAF on the viability of fibroblasts, it decreased from 85.92% to 68.32%, and from 96.21% to 46.66%, in MTT and NRU tests, respectively, with increasing NAF concentrations from 0 µg/mL to 1000 µg/mL. A statistically significant (at *p* < 0.005) decrease in cell viability was observed from 5 µg/mL, while the concentration at which the cell viability was reduced by approximately 50% was ≥500 µg/mL (Figure 22a,b), similarly to the impact of DIF. However, when the impact of NAF on the fibroblast viability was examined in the Live/Dead test, we did not observe any differences between the number of live fibroblasts in the samples irradiated in the presence of NAF (1000 µg/mL) and without NAF (0 µg/mL). It could be supposed that discrepancies between the results obtained in the MTT, NRU, and ROS tests on the one hand, and in Live/Dead test on the other hand, may have been due to the specific fibroblasts functions monitored in respective tests.

In our experiments, the phototoxic effects of DIF and NAF on the fibroblasts occurred at the lowest concentration being tested, i.e., 5 µg/mL. Thus, it would be interesting to perform subsequent tests using lower concentrations of these drugs. At the same time, the need to use various in vitro tests to better assess the effect of drugs on different cell functions was confirmed [34].

## 3. Materials and Methods

### 3.1. Materials for Chemical Tests

Diflunisal (DIF), benzoic acid, naphazoline hydrochloride (NAF) and papaverine hydrochloride (the drugs being examined and the internal standards for LC-UV methods), quinine hydrochloride, benzocaine, imidazole, p-nitrosodimethylaniline, nitroblue tetrazolium chloride and neutral red dye (reagents for ROS detection and NRU test), dimethyl sulfoxide (DMSO), ethanol, methanol, acetonitrile, formic acid and water (solvents for biological tests and chromatography) from Merck (Darmstad, Germany) or J.T. Baker (Center Valley, PA, USA) were used. Acetic acid, hydrochloric acid, phosphoric acid, sodium salts, i.e., heptanesulfonate, acetate, chloride, tetraborate, hydrogen phosphate and dihydrogen phosphate, kalium hydroxide and kalium dihydrogen phosphate (chemicals used as ingredients in mobile phases and buffers) were purchased from POCh (Gliwice, Poland).

### 3.2. Percentage Photodegradation

A chromatograph from Waters UK Sales (Elstree, UK) with a model 515 pump, a Rheodyne 20 µL injector and a model UV 2487 DAD, controlled by Empower 3 software (all from Waters) were used. Analysis was carried out on a LiChrospher^®^RP8 column (125 × 4.0 mm, 5 µm) from Merck with the mixture of 140 mL of water, 60 mL of acetonitrile, 1 mL acetic acid and 0.22 g sodium heptanesulfonate with the flow rate of 2 mL/min and UV detection for both DIF and NAF at 255 or 280 nm, respectively.

Selectivity of the methods was examined by determination of DIF and NAF in presence of their degradation products. For calibration and linearity, working solutions were prepared from the stock solutions (1 mg/mL) of DIF or NAF and respective internal standard (i.s.). The ratios of peak areas (DIF or NAF versus i.s.) against corresponding concentrations of the analyte were used for calculating calibation equations y = ax + b. The obtained SD values of the b and a coefficients values were used for determination of LOD and LOQ values. Accuracy and precision of the methods were verified at low, medium and high concentrations of DIF and NAF.

### 3.3. UPLC-HRMS/MS for Identification of Degradation Products

An ACQUITY UPLC I-Class chromatograph coupled with a Synapt G2-S HDMS mass spectrometer from Waters was applied. It was equipped with an ESI source working in a negative mode for DIF and positive mode for NAF, a q-TOF type mass analyzer and an ACQUITY UPLC BEH C18 column (2.1 × 100 mm, 1.7 µm) from Waters. Eluents A (acetonitrile) and B (formic acid in water (0.1%, *v*/*v*)) were applied at a flow rate of 0.3 mL/min using gradient conditions with over 10 min acetonitrile from 5% to 100%. The UV chromatograms were recorded at 255 or 280 nm for DIF and NAF, respectively. The following MS parameters were applied: capillary voltage 3.0 kV, desolvation gas flow 600 L/h, the temperature 250 °C, the sampling cone voltage 40 V, the source offset 80 V, and the source temperature 100 °C. The data was obtained in a scan mode ranging from 100 to 1500 *m*/*z*. Leucine-Enkephaline solution was used as the lock-spray reference material. MassLynx v4.1 software package from Waters was used for processing the recorded data.

### 3.4. Photodegradation of DIF and NAF in Solutions

Volumes of 1 mL of the stock solutions of DIF and NAF (1 mg/mL) were mixed with 1 mL of the respective solvents, i.e., 0.1 M HCl (pH 1), buffers of pH 4, 7, 10 and 0.1 M NaOH (pH 13) in quartz glass-stoppered dishes. The samples were exposed to UVA/VIS irradiation for 7–35 h with energies 18,902, 37,804, 56,706, 75,608 and 94,510 kJ/m^2^, respectively, in a Suntest CPS Plus chamber with a built-in meter from Atlas (Linsengericht, Germany) equipped with the temperature control unit set at 35 °C.

After the irradiation, the samples were neutralized if necessary, diluted with methanol to an equal volume, and analyzed quantitatively by the LC-UV methods. Concentrations of non-degraded DIF and NAF remaining after each time of irradiation were calculated from the calibration equation and the starting concentration of the analytes. The kinetic calculations were based on the linear relationships between the concentrations of non-degraded DIF and NAF remaining each time of irradiation or the logarithms of these concentrations and the corresponding time of irradiation [26]. Using our experimental data, the respective equations y = ax + b and the determination coefficients R^2^ were obtained for all the pH values, allowing the estimation of the order of reactions. Finally, they were used to calculate the rate constant (k) of photodegradation, the degradation time of 10% substance (t_0.1_), and the degradation time of 50% substance (t_0.5_).

### 3.5. ROS Monitoring

The analytical procedures were described in details in our previous work and the literature resources [22,23,24,35]. Briefly, the samples of DIF and NAF at final concentrations 20 µM were irradiated for 1–60 min (49, 234, 675, 1350, 2025, 2700 kJ/m^2^) using the Suntest CPS Plus chamber. The selected energies spanned the range of 5–20 J/cm^2^ which is recommended in the literature [22] and, additively, some higher energies. The determination of ^1^O_2_ was based on bleaching nitrosodimethylaniline in the presence of imidazole as a selective acceptor of ^1^O_2_ monitored at 440 nm while the assay of O_2_^−^ was based on monitoring reduction of nitroblue tetrazolium at 560 nm [20,21,22]. All measurements were repeated three times for each sample using a UV/VIS double beam spectrophotometer CE 6600 from Cecil Instruments Ltd. (Milton, UK).

### 3.6. In Vitro Experiments

In vitro studies on the mice BALB/c 3T3 fibroblasts from ATCC (Manassas, VA, USA) were described in detail in our previous work [35]. Briefly, for MTT and NRU tests fibroblasts were preincubated in the 96 well plates, mixed with 100 µL of DIF or NAF solutions prepared in DMSO at concentrations ranging from 0 to 1000 µg/mL. After the incubation at 37 °C for 4 h, they were irradiated through the lids with UVA/VIS light energy of 5 J/cm^2^ [22]. At the same time, the non-irradiated cells were kept in the dark. For MTT test, 10 µL of 3-(4,5-dimethylthiazol-2-yl)-2,5-diphenyl tetrazolium bromide solution (MTT) from MTT Toxicology Assay kit Tox-1KT (Merck) was added to each well. Finally, the absorbance was assayed at 560 nm using a microplate reader Tecan Sunrise controlled by the Magellan v7.1. software from Tecan Trading SA (Mannedorf, Switzerland). For the NRU test, the fibroblasts were mixed with DIF or NAF solutions, irradiated, reincubated with 100 µL of NR solution and finally, the absorbance of each well was measured at 540 nm with a microplate reader described above.

For the Live/Dead test, the fibroblasts were seeded on the glass coverslips which were then placed in small Petri dishes and cultured with DIF or NAF at a concentration of 1000 µg/mL. After the incubation and irradiation with UVA/VIS energy of 5 J/cm^2^ [22], 500 µL of Live/Dead reagent including CA-AM and EthD-1 (Live/Dead Viability/Cytotoxicity kit from Thermo Fisher Scientific, Waltham, MA, USA) was added to each dish. After reincubation, the cells were analyzed under an upright fluorescence microscope Nikon ECLIPSE Ni equipped with Nikon Plan Apo 100 × 1.45 objective and filters for FITC (excitation at 495 nm, emission at 515 nm), and Texas Red (excitation at 595 nm, emission at 615 nm) from Nikon (Tokyo, Japan) by means of GENESIS v7.0 imaging software from Applied Spectral Imaging (Carlsbad, CA, USA).

### 3.7. Statistical Analysis

Statistical analysis was performed using Statistica 13 software (TIBCO Software Inc., Pale Alto, CA, USA). For the MTT and NRU tests, the differences among the groups were assessed using one-way ANOVA followed by Tukey test.

## 4. Conclusions

In the present study, the photodegradation of two small molecule drugs with different acid–base properties, as well as their potential phototoxicity, were examined using chemical, in silico, and in vitro methods. A forced degradation showed a visible impact of pH on their photostability, with the highest degradation of acidic DIF at pH 1 and 13 and the highest degradation of basic NAF at pH below 7. In addition, four degradation products of DIF from DD-1 to DD-4 were identified by analyzing the samples irradiated at pH 4. None of these DD structures showed fragments with the risk of mutagenic, tumorigenic, irritant and reproductive effects using OSIRIS Property Explorer. However, the Toxtree software classified the compounds DD-2, DD-3 and DD-4 to the III class of high toxicity. Next, six degradation products of NAF from ND-1 to ND-6 were identified, depending on the pH used. OSIRIS Property Explorer did not show the toxicity risk for five degradation products, but the ND-2 product was shown to present a medium mutagenic and a high tumorigenic risk. In addition, Toxtree software classified all the degradation products from ND-1 to ND-6 to the III class of toxicity. Next, both DIF and NAF were observed as photoreactive and generating ROS, especially ^1^O_2_ with values higher than the cut-off treshold according to the official criteria [22]. Finally, the phototoxic effects of DIF and NAF on the fibroblast viability was observed at the lowest concentrations being tested, using the MTT and NRU tests.

It should be emphasized that the determination of photodegradation kinetics and the identification of photodegradation products of DIF and NAF were not described in the literature so far. Also, such a comprehensive study including ROS generation and in vitro tests for the potential phototoxicity were not reported for these two drugs. Therefore, we hope that our present results will be useful when designing further phototoxicity studies for DIF and NAF as well as other active pharmaceutical substances to exclude or minimize the risk of undesired effects due to their photoreactivity and photodegradation.

## Figures and Tables

**Table 1:** Quantitative LC-UV assays of diflunisal (DIF) and naphazoline (NAF).

Parameter	DIF	NAF
Linearity range (µg/mL)	20–120	10–100
Slope (mean ± SD, n = 5)	0.0393 ± 0.00011	0.0509 ± 0.00025
Intercept (mean ± SD, n = 5)	−0.0838 ± 0.00551	−0.0226 ± 0.00632
R^2^ (mean ± SD, n = 5)	0.999 ± 0.0001	0.999 ± 0.0003
LOD (µg/mL)	0.46	0.41
LOQ (µg/mL)	1.39	1.24
% Recovery (mean ± SD, n = 9)	99.5 ± 1.14	99.3 ± 1.84
% RSD for within-day precision (n = 3)	1.08	1.53
% RSD for between-day precision (n = 9)	2.37	2.12

SD = standard deviation; RSD = relative standard deviation; LOD = limit of detection; LOQ = limit of quantification.

**Table 2:** Photodegradation of diflunisal (DIF) and naphazoline (NAF) in solutions of pH 1–13.

pH	Degradation [%] and R^2^				k [s^−1^]		t_0.1_ [min]		t_0.5_ [min]	
	DIF		NAF		DIF	NAF	DIF	NAF	DIF	NAF
1	99.05	0.9712	96.47	0.9913	6.9 × 10^−3^	4.8 × 10^−3^	0.32	0.36	1.67	2.39
4	91.08	0.9924	96.15	0.8849	2.5 × 10^−3^	5.8 × 10^−3^	0.69	0.31	4.56	2.01
7	30.35	0.8285	28.54	0.7933	4.6 × 10^−4^	9.2 × 10^−5^	3.79	19.02	25.05	125.54
10	52.45	0.9282	26.03	0.9871	6.9 × 10^−4^	2.3 × 10^−5^	2.53	76.09	16.71	502.17
13	97.87	0.7272	14.74	0.9196	4.1 × 10^−3^	4.6 × 10^−5^	0.42	38.04	2.78	251.09

R^2^ = determination coefficient for linear relationships between the logarithms of concentrations of non-degraded DIF or NAF and the corresponding time of irradiation; k = the rate constant; t_0.1_ = time of degradation of 10% substance; t_0.5_ = time of degradation of 50% substance.

**Table 3:** Singlet oxygen (^1^O_2_) and superoxide radical anion (O_2_^−^) generation by diflunisal (DIF) and naphazoline (NAF) under UVA/VIS irradiation.

Compound	49 kJ/m^2^	234 kJ/m^2^	675 kJ/m^2^	1350 kJ/m^2^	2025 kJ/m^2^	2700 kJ/m^2^
Singlet oxygen ^1^O_2_						
DIF	16	54	103	143	176	455
NAF	11	45	120	238	365	480
Quinine	98	156	282	319	401	493
Benzocaine	−13	−10	−9	−8	−3	10
Superoxide anion O_2_^−^						
DIF	12	34	36	89	143	174
NAF	18	31	66	81	107	179
Quinine	81	127	133	148	179	221
Benzocaine	−13	−13	−11	−8	−6	2

Quinine = positive control; Benzocaine = negative control.

## Data Availability

Data available on request due to restrictions.
